# RETRACTION: Effect of Two Different Surgical Modalities for Pelvic Organ Prolapse on Postoperative Wound Infection in Patients: A Meta‐Analysis

**DOI:** 10.1111/iwj.70410

**Published:** 2025-03-21

## Abstract

**RETRACTION**: CuiH. , LangX. , HuangC. , and SunJ. , “Effect of Two Different Surgical Modalities for Pelvic Organ Prolapse on Postoperative Wound Infection in Patients: A Meta‐Analysis,” International Wound Journal 21, no. 3 (2024): e14802, 10.1111/iwj.14802.38472131 PMC10932775

The above article, published online on 12 March 2024, in Wiley Online Library (http://onlinelibrary.wiley.com/), has been retracted by agreement between the journal Editor in Chief, Professor Keith Harding; and John Wiley & Sons Ltd. Following an investigation by the publisher, all parties have concluded that this article was accepted solely on the basis of a compromised peer review process. The editors have therefore decided to retract the article. The authors did not respond to our notice regarding the retraction.

**RETRACTION**: H. Cui , X. Lang , C. Huang , and J. Sun , “Effect of Two Different Surgical Modalities for Pelvic Organ Prolapse on Postoperative Wound Infection in Patients: A Meta‐Analysis,” International Wound Journal 21, no. 3 (2024): e14802, 10.1111/iwj.14802.38472131 PMC10932775

The above article, published online on 12 March 2024, in Wiley Online Library (http://onlinelibrary.wiley.com/), has been retracted by agreement between the journal Editor in Chief, Professor Keith Harding; and John Wiley & Sons Ltd. Following an investigation by the publisher, all parties have concluded that this article was accepted solely on the basis of a compromised peer review process. The editors have therefore decided to retract the article. The authors did not respond to our notice regarding the retraction.

